# Detection of a Novel, Integrative Aging Process Suggests Complex Physiological Integration

**DOI:** 10.1371/journal.pone.0116489

**Published:** 2015-03-11

**Authors:** Alan A. Cohen, Emmanuel Milot, Qing Li, Patrick Bergeron, Roxane Poirier, Francis Dusseault-Bélanger, Tamàs Fülöp, Maxime Leroux, Véronique Legault, E. Jeffrey Metter, Linda P. Fried, Luigi Ferrucci

**Affiliations:** 1 Groupe de recherche PRIMUS, Department of Family Medicine, University of Sherbrooke, 3001 12e Ave N, Sherbrooke, QC, J1H 5N4, Canada; 2 Department of Chemistry, Biochemistry and Physics, Université du Québec à Trois-Rivières, 3351, boul. des Forges, C.P. 500, Trois-Rivières, QC, G9A 5H7, Canada; 3 Department of Biology, University of Sherbrooke, 2500 boulevard de l'Université, Sherbrooke, QC, J1K 2R1, Canada; 4 Department of Mathematics, University of Sherbrooke, 2500 boulevard de l'Université, Sherbrooke, QC, J1K 2R1, Canada; 5 Department of Geriatrics, University of Sherbrooke, 3001 12e Ave N, Sherbrooke, QC, J1H 5N4, Canada; 6 Economics Department, ESG, Université du Québec à Montréal, 315 rue Sainte-Catherine Est, Montréal, QC, H2X 3X2, Canada; 7 Translational Gerontology Branch, Longitudinal Studies Section, National Institute on Aging, National Institutes of Health, MedStar Harbor Hospital, 3001 S. Hanover Street, Baltimore, Maryland 21225, United States of America; 8 Mailman School of Public Health, Columbia University, 722 W. 168th Street, R1408, New York, New York 10032, United States of America; University of Wisconsin, UNITED STATES

## Abstract

Many studies of aging examine biomarkers one at a time, but complex systems theory and network theory suggest that interpretations of individual markers may be context-dependent. Here, we attempted to detect underlying processes governing the levels of many biomarkers simultaneously by applying principal components analysis to 43 common clinical biomarkers measured longitudinally in 3694 humans from three longitudinal cohort studies on two continents (Women’s Health and Aging I & II, InCHIANTI, and the Baltimore Longitudinal Study on Aging). The first axis was associated with anemia, inflammation, and low levels of calcium and albumin. The axis structure was precisely reproduced in all three populations and in all demographic sub-populations (by sex, race, etc.); we call the process represented by the axis “integrated albunemia.” Integrated albunemia increases and accelerates with age in all populations, and predicts mortality and frailty – but not chronic disease – even after controlling for age. This suggests a role in the aging process, though causality is not yet clear. Integrated albunemia behaves more stably across populations than its component biomarkers, and thus appears to represent a higher-order physiological process emerging from the structure of underlying regulatory networks. If this is correct, detection of this process has substantial implications for physiological organization more generally.

## Introduction

Most research on biomarkers of aging is conducted one molecule at a time, and indeed a number of age-related changes in biomarkers have been identified [1,2]. In some cases, there is also evidence that biomarkers, notably inflammatory cytokines, may be causally implicated in aspects of aging [2–4]. Nonetheless, there is good reason to believe that treatment of biomarkers one at a time might often be misleading, and might miss important patterns in physiological changes with age. When individual biomarkers are useful, it is precisely because they are integrated into regulatory networks and thus indicate something general about overall health state [5]. These regulatory networks are a prime example of both a complex adaptive system [6] and a directed self-organizing system [7]. Many properties and behaviors of such systems – including feedback loops, non-linear dynamics, and system-level properties – are well-known and regularly applied in fields such as physics [7] and community ecology [8], but are rarely used to understand physiology or aging (but see [9]). Such properties lead to the hypothesis that the interpretation of changes in a single biomarker will often depend on the states of other biomarkers in the system. Indeed, data from the Tsimane hunter-gatherers in Bolivia show that high levels of C-reactive protein and low levels of HDL do not have the same implications in that population that they generally do in modern Western populations [10,11].

This perspective suggests that a useful approach to studying biomarkers of aging may be to use statistical approaches that can integrate information across large numbers of biomarkers. This might allow us to characterize behaviors and properties of the system at a higher level of organization, and perhaps to understand whether and how aging may be caused by dysregulation of these networks. Indeed, some recent data suggest that aging is tightly related to dysregulatory state as measured by aberrant individual biomarker profiles [12,13]. A number of previous studies have taken various approaches to integrating biomarkers during aging, including principal components analysis (PCA) [14,15], indices of allostatic load [16,17], and calculations of biological age [18,19].

In this study, we used PCA, a statistical method to summarize multiple variables, to characterize the correlation structure of 43 biomarkers measured longitudinally in three distinct cohorts of aging humans in Europe and North America. Our objective differs from previous studies using PCA on aging biomarkers in that we are not trying to measure biological age [14,15], but rather to understand underlying physiology. We hypothesized that this approach would yield higher-level biological patterns that were not evident studying the biomarkers one at a time, and that these patterns might yield insight into the aging process more generally. The replication of analyses across distinct populations and sub-populations (by age, sex, race, etc.) allowed us to test whether any patterns detected were general or population-specific. We detected two stable, reproducible underlying processes, one novel and the other representing metabolic syndrome.

## Methods

### Data

Three longitudinal cohort studies of aging were used. The Baltimore Longitudinal Study of Aging (BLSA) is one of the world's best known, oldest, and longest longitudinal studies of aging in humans, continuing since 1958 [20,21]. Participants are community-dwelling adults in the Baltimore and Washington DC areas aged 21–96, primarily Caucasians from middle- or upper-middle-class backgrounds. Follow-up is approximately every two years. A 2003 re-design of methodology was tailored to improve the inference for systems-level questions, and we use data on 1205 individuals from after this date [21]. The Women’s Health and Aging Study (WHAS) is a population-based prospective study of community-dwelling women. Originally, WHAS was two separate studies, WHAS I including 1002 women aged 65+ among the 1/3 most disabled in the population, and WHAS II including 436 women aged 70–79 among the 2/3 least disabled. The participants were drawn from eastern Baltimore City and Baltimore County, Maryland [22]. Baseline assessment occurred from November 1992 to February 1995 in WHAS I and from August 1994 to February 1996 in WHAS II. Follow-ups were conducted roughly 1.5, 3, 6, 7.5, and 9 years later. For this study, WHAS I and WHAS II were combined into a single dataset consisting of all visits of all individuals with sufficient biomarker data for analysis. *Invecchiare in Chianti* (Aging in Chianti, “InCHIANTI”) is a prospective population-based study of 1156 adults aged 65–102 and 299 aged 20–64 randomly selected from two towns in Tuscany, Italy using multistage stratified sampling in 1998 [23]. Follow-up blood and urine samples were taken in 2001–03, 2005–06, and 2007–08.

All aspects of WHAS, InCHIANTI, and BLSA research were approved by the ethics committees at the institutions responsible for data collection, and this secondary analysis was approved by the ethics committee (*Comité d’éthique de la recherche en santé chez l’humain*) at the *Centre de recherche clinique du CHUS*, project # 11-020. Participants signed informed consent for data collection and analysis. Although the data used in these analyses cannot be freely shared due to confidentiality constraints related to human medical data, they are all available to researchers submitting an appropriate research proposal: WHAS at https://jhpeppercenter.jhmi.edu/ec_proposal/login.aspx, InCHIANTI at http://www.inchiantistudy.net/obtain_data.html, and BLSA at http://www.blsa.nih.gov/researchers.

### Biomarker selection

Biomarkers were chosen based on availability in sufficient sample size across the three studies, and to avoid obligate redundancy (for example, we used creatinine and the BUN-creatinine ratio; we therefore excluded BUN). A number of markers were available only at baseline in InCHIANTI and/or BLSA, limiting their use in trajectory analysis. Thus two different subsets of biomarkers were chosen, one with 43 markers for cross-sectional analysis and one with 34 markers for longitudinal analysis. Biomarkers and their correlations with age are shown in Table 1, organized by functional group. Table 2 shows sample sizes for individuals with the complete biomarker data needed to perform PCA. The need for a shared list of biomarkers across studies resulted in a final list that was composed nearly exclusively of markers that are (a) common; (b) used often in clinic; and (c) cheap, increasing the relevance of any results for clinical implementation. PCA cannot be performed with missing data, so only complete observations were used. In a few regression models, we performed multiple imputation (see S1 Text).

**Table 1:** Biomarkers used, their values by data set, and correlations with age.

Biomarker	System	WHAS		InCHIANTI		BLSA		Corr. with age*	
		Mean	SD	Mean	SD	Mean	SD	r	p
Hemoglobin (g/dL)	Basic blood measures	13.0	1.2	13.8	1.5	13.6	1.4	−0.14	<0.0001
Hematocrit (%)	Basic blood measures	39	4	41	4	41	4	−0.10	<0.0001
Iron (μg/dL)	Basic blood measures	80	27	85	29	89	32	−0.10	<0.0001
Red cell distribution width (RDW, %)	Basic blood measures	14.1	1.4	13.8	1.2	13.5	1.5	0.19	<0.0001
Mean corpuscular hemoglobin (MCH)	Basic blood measures	30.5	2.1	30.5	2.1	30.4	2.1	0.04	<0.0001
Mean corpuscular hemoglobin conc. (MCHC)	Basic blood measures	33.1	1.2	33.7	1.0	33.5	1.2	−0.14	<0.0001
Platelets (K/μL)	Basic blood measures	242	68	236	64	231	74	−0.11	<0.0001
Ferritin (ng/mL)	Basic blood measures	112	124	123	127	107	99	0.06	<0.0001
Red blood cell count (RBC, millions/μL)	Basic blood measures	4.26	0.43	4.53	0.47	4.50	0.48	−0.16	<0.0001
Glucose (mg/dL)	Basic blood measures	114	57	94	26	93	18	0.07	<0.0001
White blood cell count (WBC, K/μL)	Immune measures	6.3	2.4	6.3	1.7	6.0	3.5	0.10	0.32
Leukocytes (Differential Polys, %)	Immune measures	60	10	59	9	55	10	0.12	<0.0001
Monocytes (%)	Immune measures	6.9	2.4	6.6	2.2	9.2	4.3	0.10	<0.0001
Lymphocytes (%)	Immune measures	29	9	31	8	32	10	−0.18	<0.0001
Eosinophils (%)	Immune measures	3.0	2.2	3.2	2.1	3.5	2.3	0.05	<0.0001
Basophils (%)	Immune measures	0.74	0.53	0.52	0.35	0.55	0.32	0.003	0.77
Interleukin 6 (IL-6, pg/mL)†	Immune measures	4.3	9.7	3.4	2.4	2.9	2.3	0.33	<0.0001
C-reactive protein (CRP, μg/mL)†	Immune measures	6.3	8.5	4.5	9.0	3.0	5.9	0.17	<0.0001
Calcium (mg/dL)	Electrolytes	9.5	0.5	9.4	0.5	9.3	0.4	−0.03	0.001
Chloride (mEq/L)	Electrolytes	103	4	106	4	104	3	−0.03	0.004
Magnesium (mg/dL)	Electrolytes	1.99	0.20	2.08	0.36	2.05	0.20	0.05	<0.0001
Sodium (mEq/L)	Electrolytes	140.0	2.9	141.2	2.9	141.7	2.8	0.01	0.25
Potassium (mEq/L)	Electrolytes	4.2	0.43	4.19	0.40	4.20	0.34	0.10	<0.0001
Vitamin B12 (pg/mL)†	Vitamins	494	307	471	334	640	366	−0.01	0.42
Folate (W,I: nmol/L, B: ng/mL)†	Vitamins	12.4	10.4	10.1	6.9	24.6	14.2	0.09	<0.0001
IGF-1 (μg/dL)†	Hormones	119	54	129	65	125	47	−0.34	<0.0001
Estradiol (pg/mL)†	Hormones	16.2	17.8	10.1	14.8	19.6	30.7	−0.24	<0.0001
DHEAS (μg/dL)†	Hormones	43	37	104	87	64	60	−0.34	<0.0001
Thyroid stimulating hormone (TSH, mIU/L)†	Hormones	2.3	2.9	1.9	4.7	2.6	2.1	0.07	<0.0001
Total cholesterol (mg/dL)	Lipids	224	41	212	42	191	37	−0.02	0.03
Triglycerides (mg/dL)	Lipids	160	98	127	77	103	58	0.03	0.002
HDL (mg/dL)	Lipids	55	16	57	15	59	17	0.01	0.17
Albumin (W,B: g/dL; I: %)	Proteins, liver, and kidney	4.1	0.3	58.9	4.2	4.1	0.3	−0.23	<0.0001
Albumin-globulin ratio	Proteins, liver, and kidney	1.46	0.26	1.46	0.25	1.36	0.22	−0.19	<0.0001
Alkaline Phosphatase (U/L)	Proteins, liver, and kidney	87	35	165	110	78	23	0.11	<0.0001
BUN-creatinine ratio†	Proteins, liver, and kidney	19.1	5.9	37.8	10.4	16.9	4.8	0.20	<0.0001
Total proteins (g/dL)	Proteins, liver, and kidney	7.0	0.5	7.3	0.5	7.1	0.5	−0.07	<0.0001
gamma-glutamyl transpeptidase (GGT, U/L)	Proteins, liver, and kidney	31	36	27	32	30	24	0.02	0.12
Lactate dehydrogenase (LDH, U/L)	Proteins, liver, and kidney	177	35	344	75	430	163	0.16	<0.0001
Creatinine (mg/dL)	Proteins, liver, and kidney	1.01	0.36	0.92	0.27	1.02	0.29	0.12	<0.0001
Uric acid (mg/dL)	Proteins, liver, and kidney	5.6	1.7	5.2	1.4	5.3	1.4	0.12	<0.0001
Alanine transaminase (ALT, U/L)	Proteins, liver, and kidney	19.6	10.9	20.8	10.5	32.0	12.4	0.05	<0.0001
Aspartate transaminase (AST, U/L)	Proteins, liver, and kidney	16.2	12.1	19.4	15.2	28.1	10.6	−0.14	<0.0001

*Measured based on the combined data after transformation for normality and standardization by sex and scale.

†The variable was not used in the set of 34 with larger sample size, only in the set of 43.

**Table 2:** Sample sizes for complete observations with the two variable subsets in the three data sets.

	43 variables	34 variables
**WHAS**		
Individuals	1031	1184
Visits	1849	2455
**InCHIANTI**		
Individuals	1047	1305
Visits	1047	3564
**BLSA**		
Individuals	293	1205
Visits	304	2643

### Demographic and health status variables

Age at each visit was calculated in years (decimal format to the nearest day). Demographic variables were used to stratify the samples within each dataset: for WHAS, race (white or black), married or not, more or less than 10 years education; for InCHIANTI, sex, village (Greve in Chianti or Bagno a Ripoli), and age (<65, 65–80, 80+); and for BLSA, sex, race, and age (<65, 65–80, 80+).

Health status measures were not available for BLSA. Type and availability of data varied substantially between WHAS and InCHIANTI; details are available in S1 Text. Broadly speaking, chronic disease status was available at each visit for InCHIANTI but only at baseline for WHAS, based on self-report. Frailty, measured using the phenotype definition and Fried’s frailty criteria [24], was only available at baseline for InCHIANTI but at all visits for WHAS. We examined cancer, cardiovascular disease (CVD), and diabetes, as well as a score for the number of comorbidities that ranged from 0–13 for InCHIANTI and from 0–9 for WHAS.

### Data analysis

All analyses were performed in R v3.0.1 [25]. All code is available upon request. All biomarkers were transformed before analysis. Estradiol was stratified by sex for transformation, such that each individual had a score relative to members of their sex. Then, variables were log- or square-root- transformed as necessary to approach normality. Third, all variables were centered at zero and divided by their standard deviation such that all means were 0 and all standard deviations were 1. Estradiol was then recombined. Transformations were conducted separately for each dataset.

### Principal components analysis (PCA)

PCA is a common data reduction method that can be used to characterize the correlation structure of a set of variables [26]. It identifies linear combinations of the original variables (“axes”) such that the first axis explains as much as possible of the total variance in all the variables, the second as much as possible of the remaining variance, etc., with the constraint that each axis be orthogonal to all preceding axes. The number of axes is thus equal to the number of original variables, but in most cases most of the axes are unimportant, with the first several explaining most of the variance in the system. Axes can be interpreted based on their “loadings,” which indicate the strengths of their associations with the original variables. While the most common application of PCA is for data reduction – to reduce the number of variables when there are many redundant variables – PCA can also be used to identify underlying processes that may simultaneously determine the levels of many variables, but may not be directly measurable [27,28].

PCA is not subject to standard tests of statistical significance such as *p*-values and confidence intervals, and thus other methods are needed to show that results are not the result of sampling error. We used replication to achieve this, and we believe more generally that replication is a key and underused aspect of scientific validity more generally. We applied this approach at two levels to validate PCA axis structure: first, treating population subsets (by sex, race, education, etc.) as independent populations with which to verify results, and second, using the three whole datasets for validation. If we could consistently detect the same axis across populations and sub-populations, we would have strong support for the hypothesis that the axis represents an underlying biological process; if we could not, the interpretation would be uncertain. But how do we know if we detect the same axis, given normal statistical variation? We take two approaches to answering this question. First, if the axes generated from independent datasets (and applied back to the full dataset via their loadings, see S1 Text) are consistently correlated at *r* > 0.9, they are essentially measuring the same phenomenon. Second, if the loadings of the biomarkers on the axes are consistent in strength and rank, this confirms a consistent biological interpretation. If many variables from different physiological systems have strong loadings on the same axis, this indicates that the process measured by the axis involves multiple systems.

### Trajectory analysis

Trajectories of PCA1 with age were estimated for each dataset using Bayesian mixed models implemented in R using the MCMCglmm package [29]. Due to the lack of longitudinal data in InCHIANTI and BLSA with the 43-variable set, the 34-variable set was used for principal analyses. Models were optimized to include, as necessary, population- and individual-level intercept, slope, and quadratic terms. Inclusion of individual-level terms had little effect on fixed effects; in all cases, an individual intercept was retained and individual slopes and quadratic effects were eliminated from models. Different covariance structures were included for individual-level terms; uncorrelated models converged better but produced similar parameter estimates to those with unstructured covariance. Burn-in, thinning, and number of iterations were adjusted to arrive at an MCMC sample of 1000 retained iterations with minimized auto-correlations (<0.1) after model convergence. Final analyses presented are with the autocorrelation < 0.1 between retained iterations. Sensitivity analyses showed highly similar final parameter estimation regardless of covariance structure, priors (within the generally uninformative range), parameter starting values, and thinning parameters, as well as with the full 43 variables. For the full 43 variable analysis of InCHIANTI, given that each individual was only present once in the dataset with full data, we used a linear model with slope and quadratic effects implemented in glm rather than MCMCglmm.

### Prediction of health status

For InCHIANTI and WHAS, for which we had access to health status information, we ran various regression models to predict health status based on PCA axes controlling for age. We did not control for additional covariates because the notion of causality in complex dynamic systems does not apply in the same way it does to simple, deterministic systems [30]; accordingly, we were not attempting to show even putative causal relationships between PCAs and health outcomes, but rather consistent and strong physiological associations between the process(es) represented by PCA(s) and the health outcomes.

The differences in population composition and health status measurement procedures necessitated a series of models in order to perform apples-to-apples comparisons. For example, all InCHIANTI analyses were duplicated, once on the full population and once on the subset of women aged 65+ (equivalent to WHAS). Additionally, for WHAS but not InCHIANTI we were able to predict frailty at subsequent visits rather than simply observe associations at baseline, and for InCHIANTI but not WHAS this was true for chronic diseases.

The relationship between PCA axes and mortality was assessed using time-to-event Cox proportional hazards models with age as the timescale. All visits were included as separate observations, with individuals censored at the subsequent visit, if any. (Unlike standard regression models, Cox models are not biased by the non-independence of visits nested within individuals.) Frailty criteria and number of comorbidities were assessed using both linear and Poisson regression. Although the Poisson regression is technically more appropriate, the results are similar and the linear regression coefficients are easier to interpret; it is thus the linear regression results that are presented in figures. Logistic regression was used for individual chronic diseases, with ambiguous CVD and diabetes diagnoses in InCHIANTI assigned to the positive category. (Sensitivity analyses assigning them to the negative category or using linear regression on the 0–0.5–1 scale produced comparable results.) All regression models controlled for age. Because proper control for age was essential, a linear treatment might have been insufficient. Accordingly, we used a flexible cubic basis spline (bs function, fda package, R) with four knots for WHAS or five knots for InCHIANTI to control for age more completely. (The difference in knot number was due to the larger age range in InCHIANTI; in the end, essentially identical results can be obtained with linear control for age, so details of spline specifications are not important.) All regression models other than survival models were implemented in the MCMCglmm package [29], with individual as a random effect as necessary and with uninformative priors. Results were nearly identical using the lme package for mixed effects models in a non-Bayesian framework (data not shown). Additionally, to ensure that results were not biased due to lack of proper control for age, targeted analyses were re-run on PCAs generated from biomarkers that had already been adjusted for age using a spline.

We performed a very large number of statistical tests relating PCAs to health outcomes (252 models presented in S1 Results). In such a situation, multiple testing is a potential concern. There are many schools of thought on how to deal with multiple testing; in this case, a straightforward Bonferroni correction would be inappropriate because many of the tests are redundant and correlated. The 252 regression models boil down to 18 underlying hypotheses (3 PCA axes that may or may not be related with any of 6 health outcomes). Each of these hypotheses is tested independently twice (once in WHAS, once in InCHIANTI). The additional models are alternative ways to model these questions using the same data. With 36 tests, we should expect about one or two to be significant at *p* = 0.05 just by chance. We feel the best approach to considering multiple comparisons in this case is to interpret the results in light of the number of false positives expected, the *p*-values observed, and the ability to replicate the same results across the two data sets.

### Additional analyses

Stability of individual biomarker roles across datasets was assessed by calculating Pearson correlation coefficient with age for each biomarker and the first three PCA axes in each dataset or subset. Likewise, correlations among key biomarkers associated with PCA1 were calculated in each dataset to compare consistency. In order to avoid presenting many anemia-related markers, most of which were strongly correlated, we summarized anemia via scores on the first axis of a PCA analysis of these markers, which loaded with the individual markers as expected.

Comparison of sub-population differences to differences among random samples was carried out by generating 10 random, mutually exclusive subsets of the 3200 complete observations for the 43 variables. We ran PCA on each subset, reapplied the loadings for the first axis to the full dataset, and calculated the 45 pairwise correlation coefficients between these ten alternative versions of PCA1 calculated based on random subsets. This was repeated 100 times. If the correlations among datasets and subsets are as strong as those observed among the random subsets, this indicates that random sampling alone is sufficient to account for differences among populations and sub-populations. Additionally, because the observations are not sorted by visit, this analysis provides a modest check on potential recruitment bias.

Hepcidin was log-transformed before analysis. The correlation between PCA1 and hepcidin was calculated based on the InCHIANTI-specific PCA1. A simplified, clinically feasible alternative version of PCA1 was calculated by trial and error eliminating biomarkers that were expensive, clinically difficult, or weakly loaded on PCA1, while conserving biomarkers from a variety of functional groups or those that are measured together in a standard blood panel. A user-friendly calculator for PCA1 was implemented in Excel.

## Results/Discussion

### Identification of the correlation structure among human biomarkers through principal components analysis (PCA)

**Fig 1:**
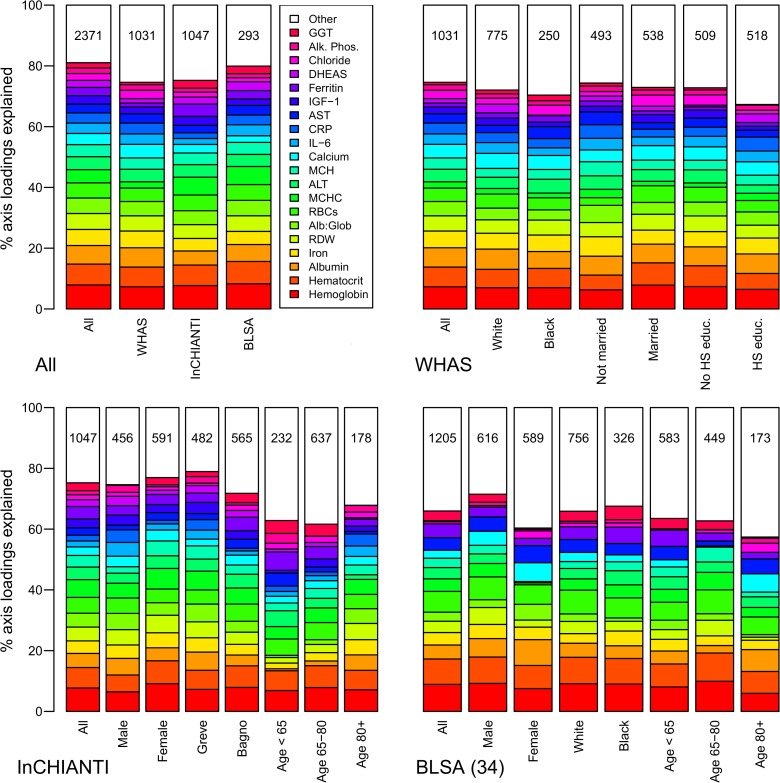
Biomarker loading order and stability for PCA1 across datasets and subsets. Loading importance is calculated as the loading divided by the sum of the absolute values of all loadings. These values are ordered from high (red, on bottom) to low (magenta, on top) for the first 20 loadings; remaining loadings are grouped together as “Other” in white. Accordingly, hemoglobin has the strongest loading, then hematocrit, then albumin, etc. The order and colors are derived from the full analysis combining the first visits of individuals in all three datasets (top-left panel, left column, “All”) and applied to all other columns in the figure. Stability of loadings is indicated by conservation of loading heights across bars. (For an example of unstable loadings, see Fig. 5.) For each panel, the loadings for the full dataset are at left. Numbers indicate subset sample sizes. For all panels except BLSA, the 43-variable set is used; for BLSA there was insufficient sample size to perform PCA on subsets with 43 variables, so the 34-variable analysis is presented.

Using PCA, we identified the correlation structure among the 43 markers (Table 1). The first axis (PCA1) explained 10.1% of the variance, 4.3 times the standard threshold for an informative axis and a surprisingly high percentage given the large total variance across all the physiological systems represented [31]. A high score on PCA1 for an individual was associated with high levels of anemia-related markers (hemoglobin, hematocrit, iron, MCHC, etc.) and the inflammatory markers CRP and IL-6, simultaneously with low levels of albumin and calcium (Fig. 1, which also can be used to infer the demographic composition of the study cohorts).

**Fig 2:**
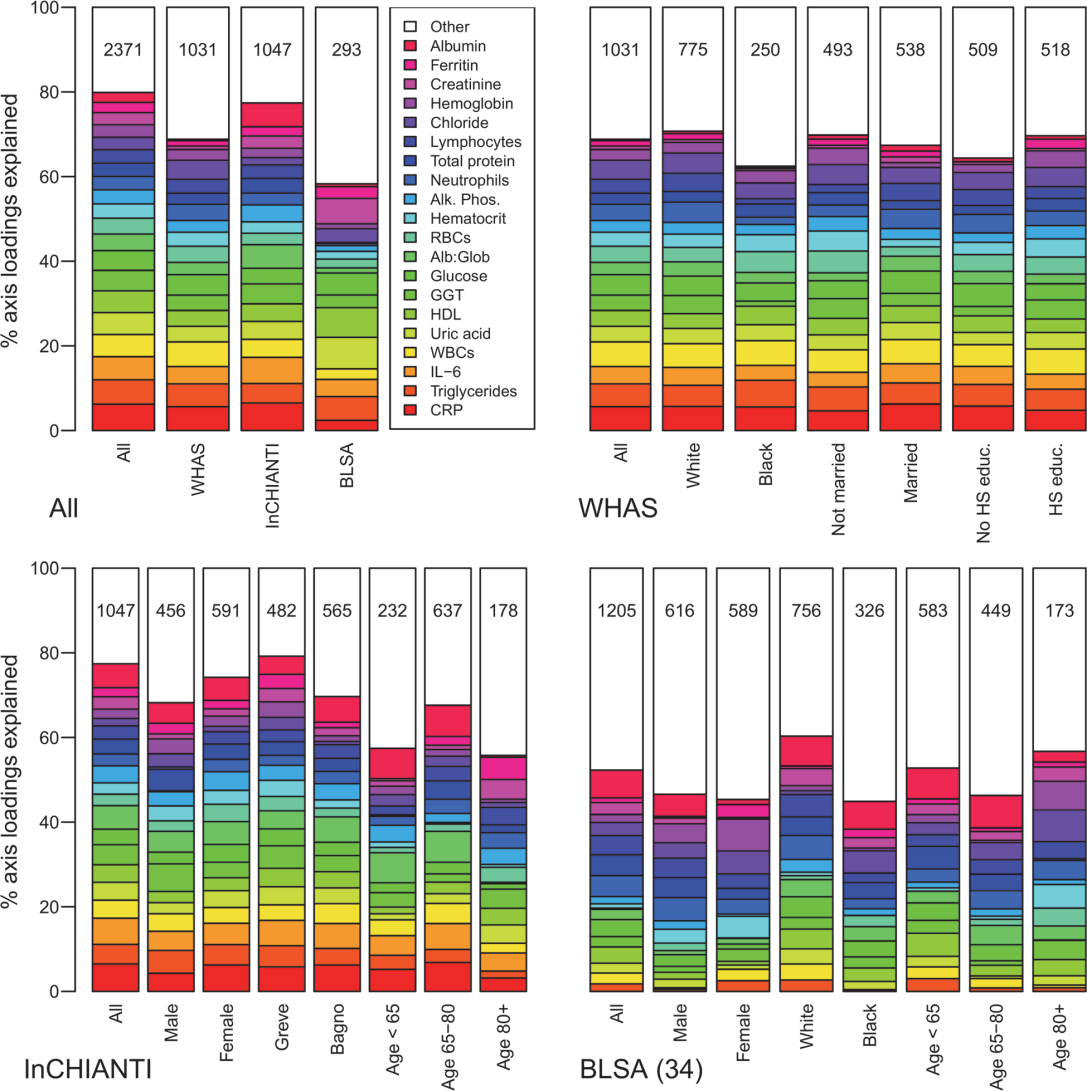
Biomarker loading order and stability for PCA2 across data sets and subsets. Loading importance is calculated as the loading divided by the sum of the absolute values of all loadings. These values are ordered from high (red, on bottom) to low (magenta, on top) for the first 20 loadings; remaining loadings are grouped together as “Other” in white. Accordingly, CRP has the strongest loading, then triglycerides, then IL-6, etc. The order and colors are derived from the full analysis combining the three data sets (left column, top-left panel “All”) and applied to all other columns in the figure. Stability of loadings is indicated by conservation of loading heights across bars. For each panel, the loadings for the full data set are at left. Numbers indicate subset sample sizes. For all panels except BLSA, the 43-variable set is used; for BLSA there was insufficient sample size to perform PCA on subsets with 43 variables, so the 34-variable analysis is presented. The poor performance of BLSA is because several of the key variables in PCA2 are missing in the 34-variable set.

**Fig 3:**
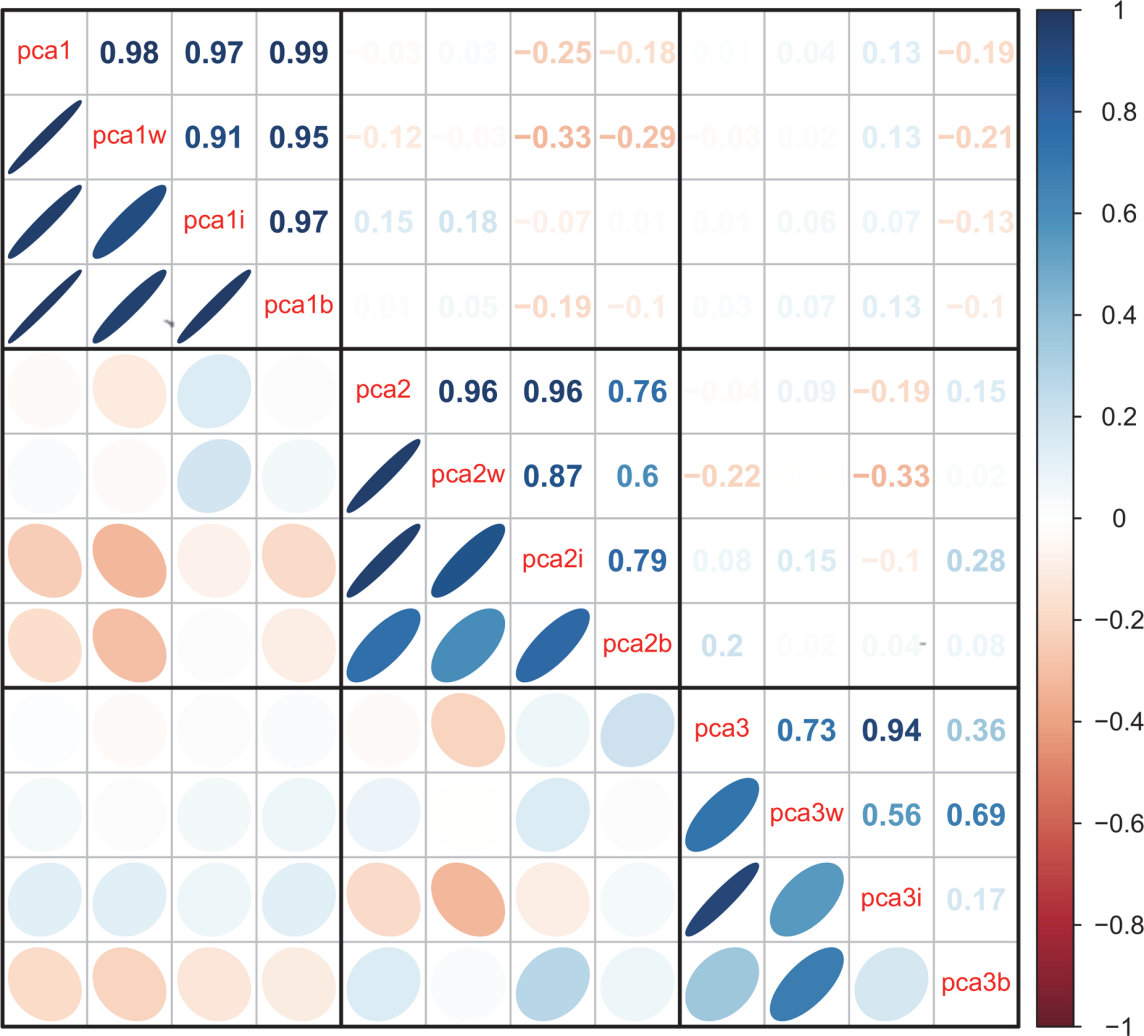
Correlations among the first three PCA axes (“pca1”, “pca2”, and “pca3”). Correlations were calculated from the full merged 43-variable data set or from each of the separate data sets (WHAS: w; InCHIANTI: i; BLSA: b). The loadings from each subset-based PCA are reapplied to the full data set to calculate the scores used in the correlations. Ellipses below the diagonal indicate correlations visually: blue when positive, red when negative, and darker and narrower when stronger. Correlation coefficients are above the diagonal.

**Fig 4:**
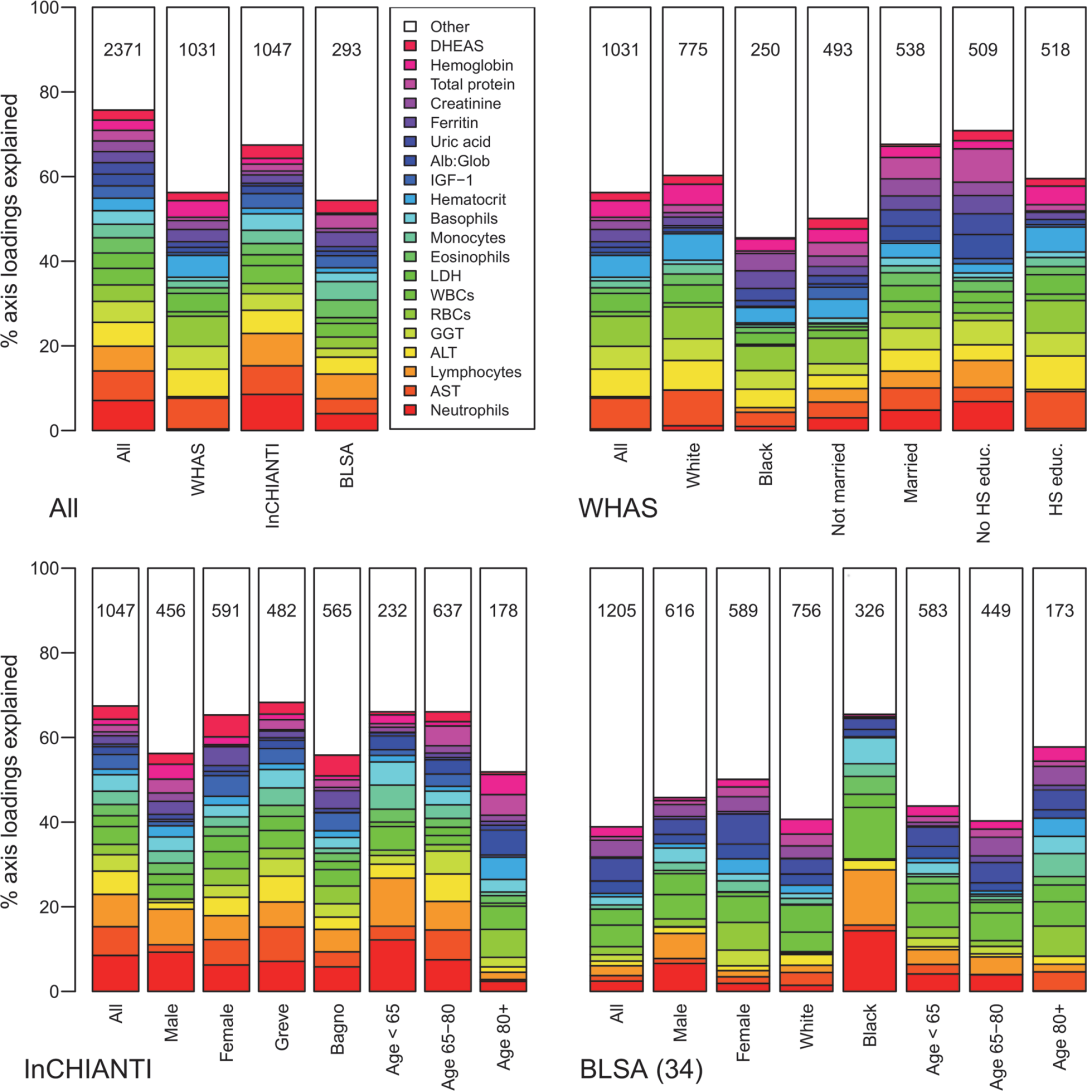
Biomarker loading order and stability for PCA3 across data sets and subsets. Loading importance is calculated as the loading divided by the sum of the absolute values of all loadings. These values are ordered from high (red, on bottom) to low (magenta, on top) for the first 20 loadings; remaining loadings are grouped together as “Other” in white. Accordingly, neutrophils have the strongest loading, then AST, then lymphocytes, etc. The order and colors are derived from the full analysis combining the three data sets (left column, top-left panel “All”) and applied to all other columns in the figure. Stability of loadings is indicated by conservation of loading heights across bars. For each panel, the loadings for the full data set are at left. Numbers indicate subset sample sizes. For all panels except BLSA, the 43-variable set is used; for BLSA there was insufficient sample size to perform PCA on subsets with 43 variables, so the 34-variable analysis is presented.

**Fig 5:**
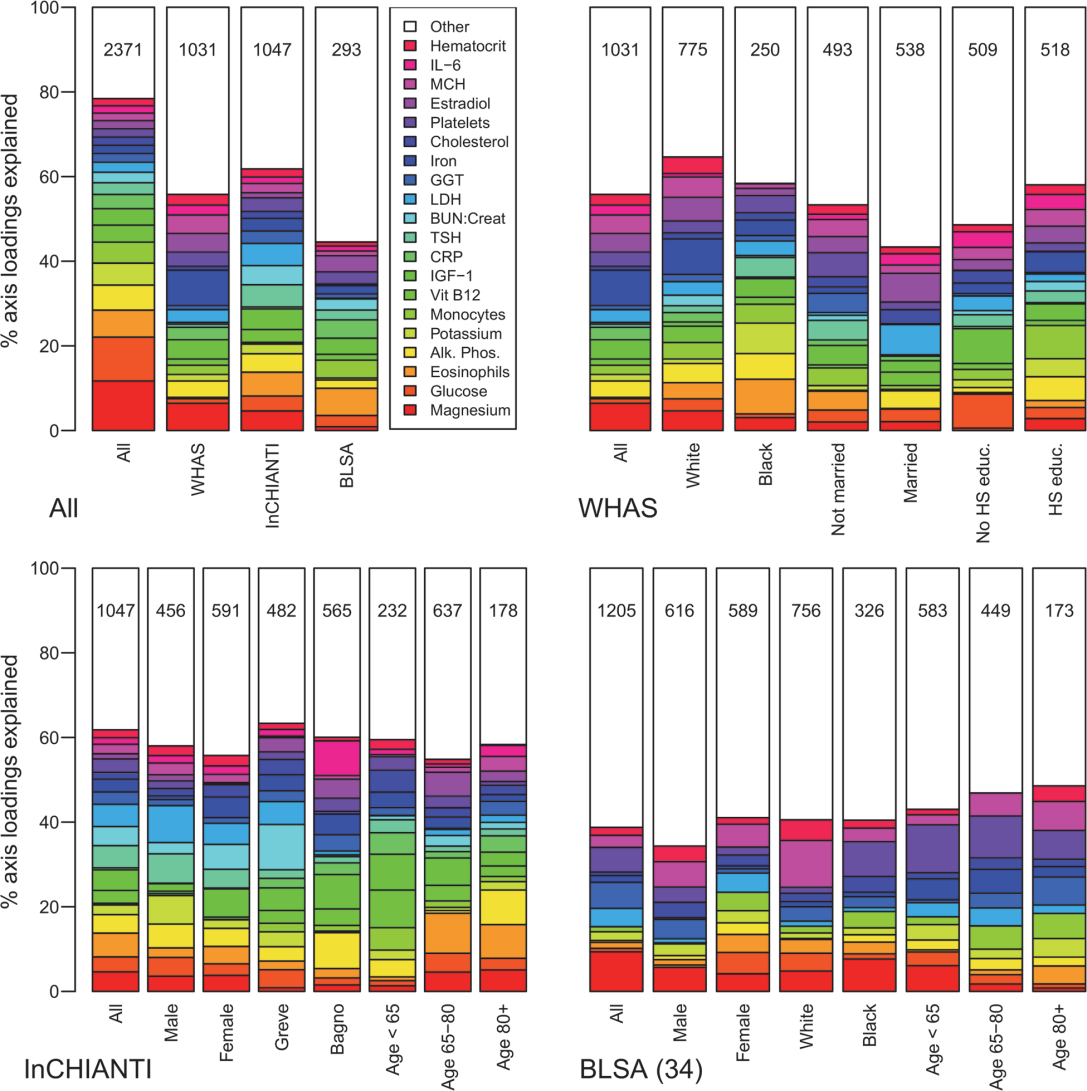
Biomarker loading order and stability for PCA25 (the 25^th^ axis, chosen randomly as an example of an unstable axis) across data sets and subsets. Loading importance is calculated as the loading divided by the sum of the absolute values of all loadings. These values are ordered from high (red, on bottom) to low (magenta, on top) for the first 20 loadings; remaining loadings are grouped together as “Other” in white. Accordingly, magnesium has the strongest loading, then glucose, then eosinophils, etc. The order and colors are derived from the full analysis combining the three data sets (left column, top-left panel “All”) and applied to all other columns in the figure. Stability of loadings is indicated by conservation of loading heights across bars. For each panel, the loadings for the full data set are at left. Numbers indicate subset sample sizes. For all panels except BLSA, the 43-variable set is used; for BLSA there was insufficient sample size to perform PCA on subsets with 43 variables, so the 34-variable analysis is presented.

The second axis (PCA2) explained 7.8% of the variance and was relatively strongly correlated across datasets (Figs. 2–3), though less so than PCA1. PCA2 was strongly associated with inflammation, lipid levels, and blood sugar levels (Figs. 2–3), implying it may be a proxy for metabolic syndrome. The third axis (PCA3, Fig. 4) explained 5.5% of the variance and, while performing much better than many subsequent axes (see example of PCA25, Fig. 5) was much less stable than PCA1 and PCA2. Subsequent axes were highly unstable and are not considered further.

### Axis validation and effect of population structure on PCA1 biology

**Fig 6:**
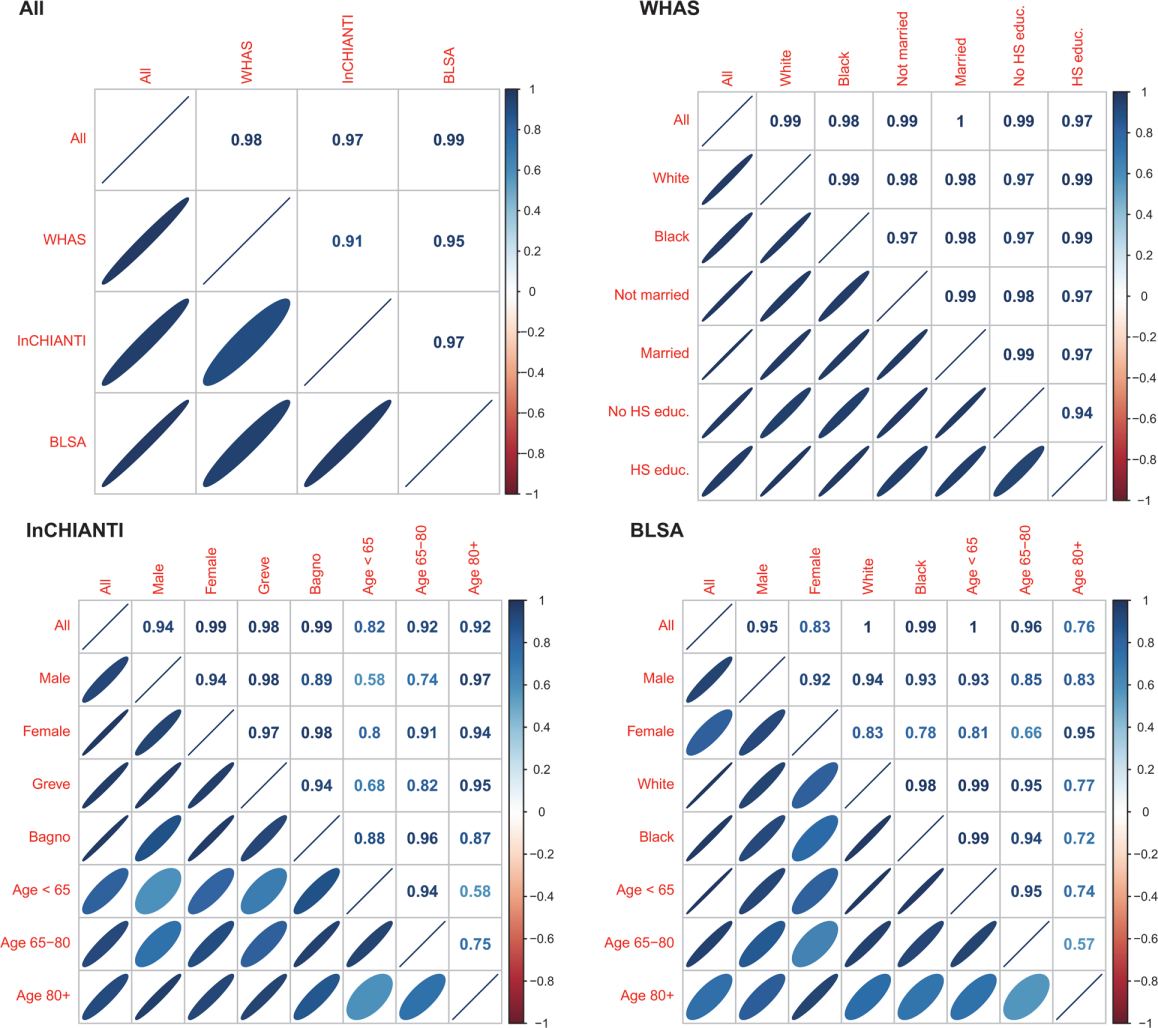
Correlations among versions of PCA1 calculated from the full merged 43-variable dataset or from data subsets. The loadings from each subset-based PCA are reapplied to randomly selected visits of the full dataset to calculate the scores used in the correlations [27,28]. Ellipses below the diagonal indicate correlations visually: blue when positive, red when negative, and darker and narrower when stronger. Correlation coefficients are above the diagonal. For all panels except BLSA, the 43-variable set is used; for BLSA there was insufficient sample size to perform PCA on subsets with 43 variables, so the 34-variable analysis is presented.

**Fig 7:**
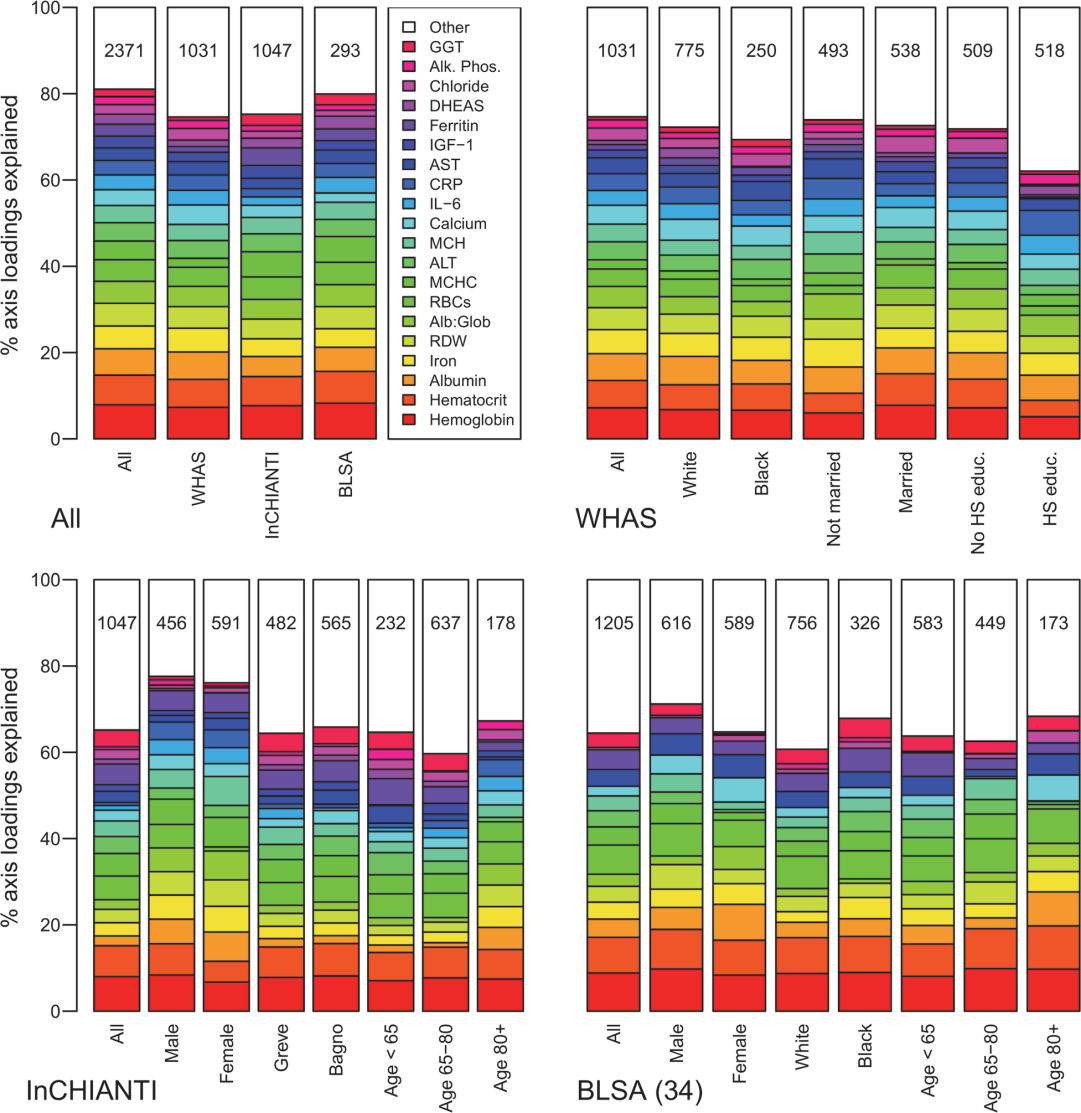
Age-adjusted biomarker loading order and stability for PCA1 across data sets and subsets. Loading importance is calculated as the loading divided by the sum of the absolute values of all loadings. These values are ordered from high (red, on bottom) to low (magenta, on top) for the first 20 loadings; remaining loadings are grouped together as “Other” in white. Accordingly, hemoglobin has the strongest loading, then hematocrit, then albumin, etc. The order and colors are derived from the full analysis combining the first visits of individuals in all three data sets (top-left panel, left column, “All”) and applied to all other columns in the figure. Stability of loadings is indicated by conservation of loading heights across bars. For each panel, the loadings for the full data set are at left. Numbers indicate subset sample sizes. For all panels except BLSA, the 43-variable set is used; for BLSA there was insufficient sample size to perform PCA on subsets with 43 variables, so the 34-variable analysis is presented.

Stability and validity of the axes were tested by replicating the analyses across populations and on subgroups of the three populations by demographic traits (e.g., sex, race, income, age; Figs. 1, 2, 4–6) [27,28]. For example, if the first axis generated by an analysis of just the men is nearly identical to that generated by an analysis of just the women, this supports the hypotheses that (a) the axis represents a real biological process rather than noise, and (b) the process does not differ by sex. When comparing versions of PCA1 generated from multiple independent datasets or subsets, correlation coefficients were uniformly very high, usually greater than 0.9 (Figs. 3, 6). Similarly, biological interpretation of PCA1 was nearly identical across datasets and subsets based on the importance of the loadings of the raw variables (Fig. 1). Additionally, PCA1 was reproduced using age-adjusted levels of biomarkers rather than raw levels (Fig. 7), showing that the characteristic PCA1 biomarker signature can be extracted even from individuals of similar age. Together, these findings show that we are detecting the same PCA1 in multiple distinct populations: Italy and the USA, men and women, blacks and whites, rich and poor, etc.

We tested the sensitivity of PCA1 to the number and composition of markers in the dataset by using three different groups of markers: 43 markers present in all three datasets with a sufficient cross-sectional sample size, 34 markers with a sufficient longitudinal sample size, and a reduced set of 14 markers for clinical application. Like the 43-variable version, the 34-variable PCA1 was highly stable (mean correlation coefficients of 0.97, 0.92 and 0.87 among PCA1 generated from data subsets of WHAS, InCHIANTI and BLSA, respectively). The 43- and 34-variable versions were strongly correlated (*r* = 0.91, *p*<0.0001), but the association was stronger still between the 43-variable version and the 14-variable clinical version (*r* = 0.97, *p*<0.0001), which uses a small number of cheap markers easily measured in clinic (hemoglobin, hematocrit, MCH, MCHC, RDW, RBC, platelet count, albumin, albumin-globulin ratio, calcium, CRP, ALT, iron, and alkaline phosphatase).

Next, we tested whether the slight differences in PCA1 across populations and sub-populations might be due to biological or sociological differences, as opposed to questions of sampling. We divided the data into 10 equal, exclusive groups and calculated the 45 correlation coefficients among PCA1s as generated in each subset. Repeating this 100 times, the mean of the mean correlation coefficients was 0.945, and the mean of the minimum correlation coefficients was 0.801. The mean interquartile range was 0.934–0.975. Comparison of these correlations to Fig. 6 shows that random sampling effects appear to be sufficient to explain most of the differences in PCA1 across populations and sub-populations. While this does not prove the complete absence of biological differences across populations, it suggests that any biological differences are sufficiently minor that we are not able to detect them even with sample sizes in the thousands.

The biomarker composition of PCA1 suggested that it could have been driven by the peptide hormone hepcidin [32,33], which regulates iron levels and appears to be associated with inflammation [34,35], albumin [36], and calcium [37]. However, one study using InCHIANTI data found no relation between urinary hepcidin and inflammation [38]. Using these same data, we found a correlation of *r* = −0.14 (*p* = 0.002) between urinary hepcidin and PCA1 among the 485 individuals with both measures. PCA1 is more strongly correlated with 23 of the 43 original markers (|*r*| > 0.14), so PCA1 does not appear to be a surrogate measure of hepcidin function.

In characterizing PCA1, highly similar results were obtained with 43-, 34-, and 14-marker analyses, indicating that detection of PCA1 is not an artifact of biomarker choice, nor particularly sensitive to the exact composition of markers in an analysis. Obviously, it would be possible to choose sets of markers in which PCA1 could not be detected (for example, by excluding all markers of anemia and inflammation). Notably, the 34-variable set – chosen based on the availability of markers for longitudinal analysis, and therefore lacking the inflammatory markers CRP and IL-6.–.was less well associated with the 43-variable PCA1 than was a 14-variable set chosen to replicate the 43-variable analysis as closely as possible in clinical settings. The stability of the axis regardless of precise biomarker choice or measurement protocols suggests that it can be measured consistently across studies that may not have access to exactly the same raw data. Indeed, results would be unlikely to change substantially if we went beyond peripheral human blood markers and used animal models and/or high-throughput genetic, epigenetic, expression, proteomic and metabolomics biomarkers [39–41]. Such analyses could help identify other physiological systems associated with PCA1, but should not affect the associations shown here. Given that PCA identifies linear combinations of biomarkers that covary together having maximal variation in a population, analyses of other biomarker sets may yield other dimensions yet to be discovered on which human populations vary even more than PCA1.

### PCA1 and aging

**Fig 8:**
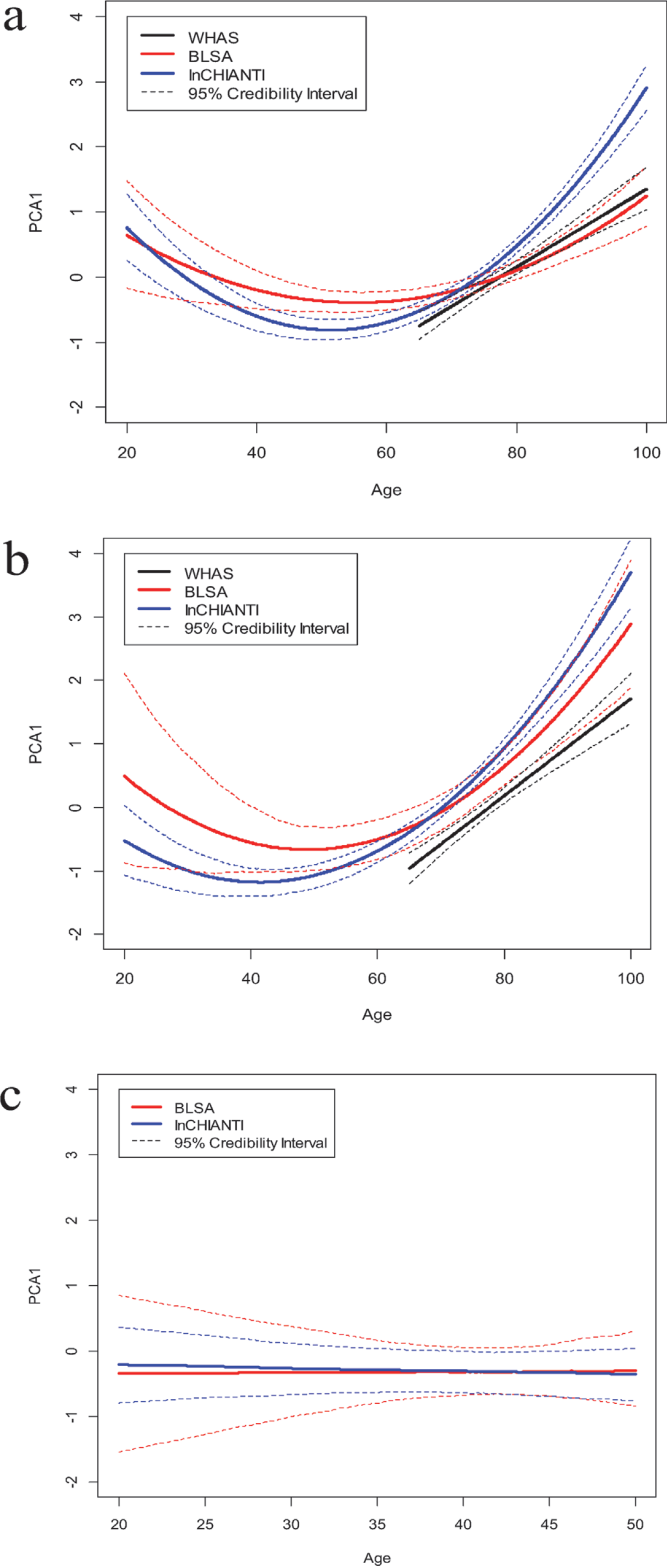
Age trajectories of PCA1 in the three data sets, based on Bayesian mixed models. (a) 34-variable data set; (b) 43-variable data set; (c) ages 20–50 only. In (a) and (b), BLSA and InCHIANTI are based on fixed quadratic models with a random (individual) intercept, while WHAS is based on a fixed linear model with a random intercept. Linear models with random intercept were used in (c). Credibility intervals are based on calculating, independently for each age, which of the 1000 iterations’ trajectories were in the 2.5^th^ and 97.5^th^ percentiles. Note that the better fit of the linear model for WHAS appears to be due to the more limited age range for this dataset.

We assigned a score for PCA1 to each individual at each data collection point. We estimated average individual age trajectories of these scores using Bayesian mixed quadratic models. Results were again concordant across datasets and show quadratic or exponential increases in levels of PCA1 with age (Fig. 8, Table 3). There was generally good support for variation across individuals in intercept (standard deviation between 2.57 and 2.92 in well-converged models) but not slope or quadratic effect. 34- and 43-variable sets gave generally concordant trajectories (Fig. 8, Table 3), though increases with age were perhaps steeper in the 43-variable set, concordant with the inclusion of inflammatory markers leading to a better measure of an aging-associated process.

**Table 3:** Results of Bayesian mixed trajectory models of PCA1 with age, best models.

	Thin	Autocorr		Fixed intercept			Fixed slope			Fixed quadratic			Random intercept	
			Beta	LCI	UCI	Beta	LCI	UCI	Beta	LCI	UCI	St. Dev.	LCI	UCI
**43 variables**														
WHAS	20	0.013	-6.04	-7.31	-4.71	0.07	0.06	0.09	-	-	-	2.87	2.55	3.16
InCHIANTI	1000	0.969	1.26	0.11	2.47	-0.12	-0.16	-0.08	0.0014	0.0010	0.0017	0.88	0.00	3.33
InCHIANTI*	-	-	1.24	0.15	2.46	-0.12	-0.16	-0.07	0.0014	0.0010	0.0018	-	-	-
BLSA	1000	0.069	2.69	-0.23	5.89	-0.14	-0.24	-0.04	0.0014	0.0005	0.0021	2.92	1.95	3.76
**34 variables**														
WHAS	10	−0.031	-4.41	-5.86	-3.45	0.06	0.05	0.08	-	-	-	2.57	2.30	2.84
InCHIANTI	20	−0.049	3.26	2.37	4.43	-0.17	-0.19	-0.12	0.0016	0.0013	0.0019	2.70	2.46	2.96
BLSA	10	0.042	1.76	0.46	3.98	-0.10	-0.14	-0.03	0.0008	0.0004	0.0012	2.71	2.45	2.99

LCI and UCI are 95% Bayesian credibility intervals based on the posterior distribution. Betas are based on the posterior mode. Autocorr: minimum autocorrelation between iterations after thin.

*: This is a cross-sectional likelihood-based glm model, not a Bayesian glmm model, because each individual appears just one time and the above Bayesian model thus converges poorly, unable to estimate individual effects. The two models nonetheless give nearly identical parameter estimates.

In both BLSA and InCHIANTI there was strong support for a positive quadratic term, indicating faster increases at older ages; in WHAS there was a clear lack of support for a quadratic term (Table 3). This difference is probably because of the larger age ranges in BLSA and InCHIANTI, where the youngest individuals are in their 20s, as opposed to 65 in WHAS. When plotted together, the trajectories of the three data sets are largely concordant (Fig. 8); nonetheless, there is a possibility that recruitment bias and/or cohort effects might have affected the observed trajectories [42]. The apparent decline in PCA1 at younger ages in BLSA and InCHIANTI (the left tail of the J-shape, Fig. 8A-B) is likely an artifact of our modeling procedure: data are sparse at these ages, and a quadratic term, in order to produce acceleration at later ages, needs to induce a curvature at the younger ages as well. Using only younger individuals from BLSA and InCHIANTI, it is evident that PCA1 levels do not actually decline earlier in life, but are essentially flat (Fig. 8C).

**Fig 9:**
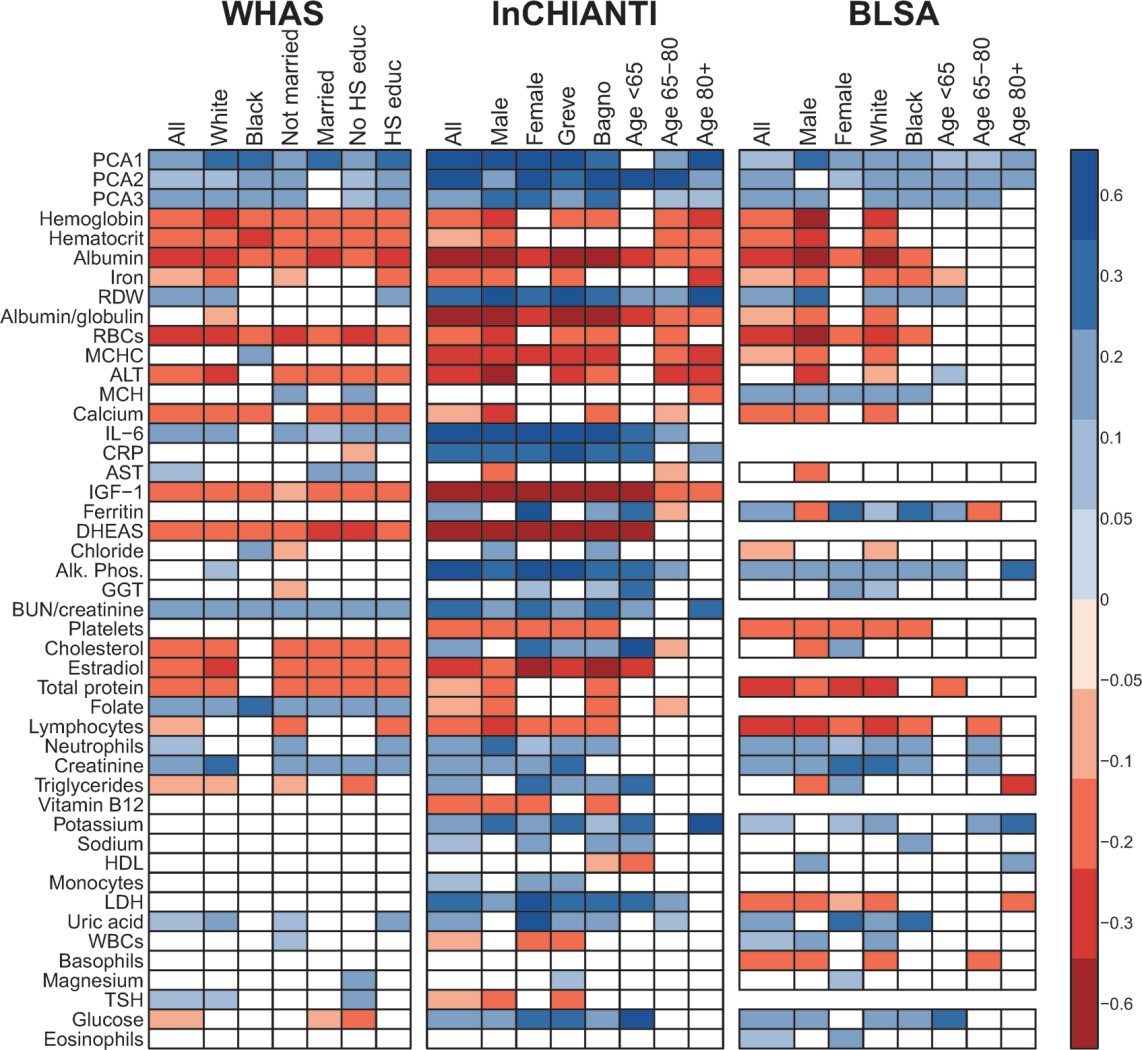
Stability across datasets of correlations between age and PCA axes or biomarkers. Biomarkers are sorted from highest to lowest |loading| in PCA1. Colored boxes indicate significant correlations (*p*<0.05) with darker shading indicating stronger correlation (blue = positive, red = negative). Note that many biomarkers are significantly correlated in opposite directions in different datasets. Boxes are absent for variables with insufficient sample size in the respective dataset. The weak correlation between age and PCA1 in the youngest subgroup is expected given the non-linear relationship with age (Fig. 8).

**Fig 10:**
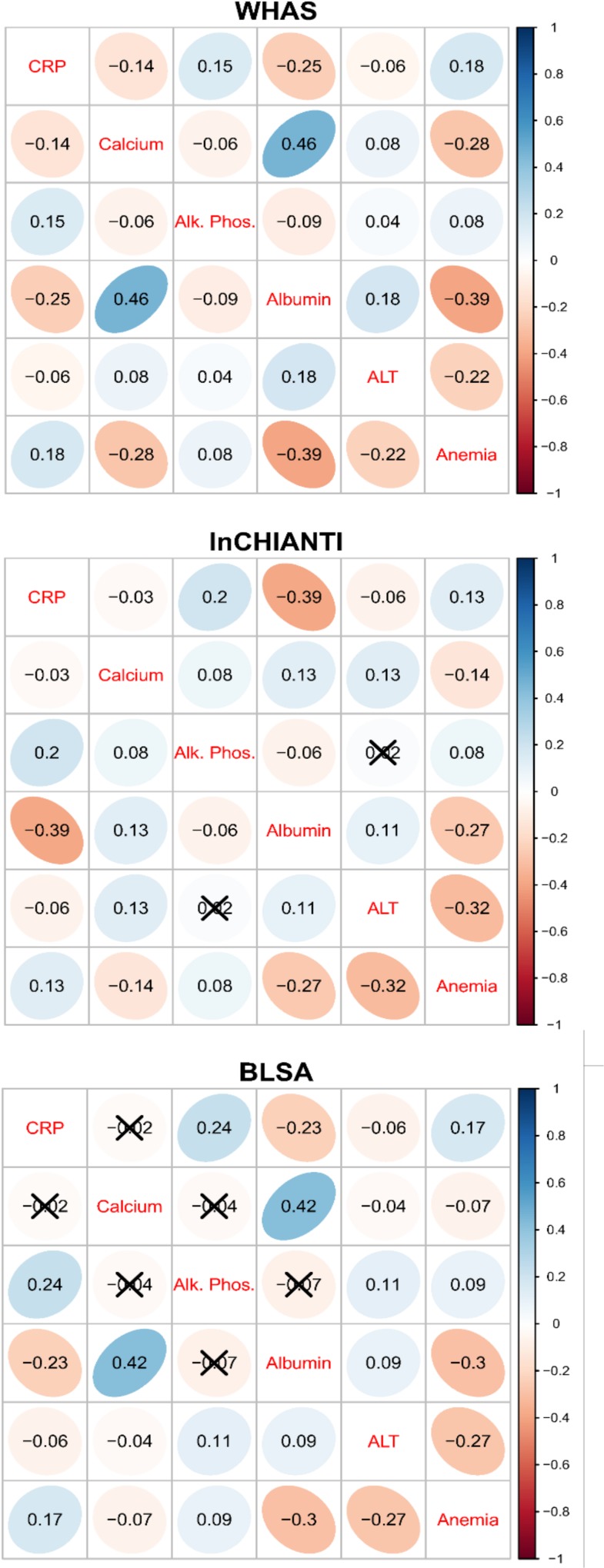
Cross-dataset comparison of pairwise correlations among the main biomarkers determining PCA1. Anemia is based on the first axis of a PCA analysis of anemia-related markers. Ellipses indicate correlations visually: blue when positive, red when negative, and darker and narrower when stronger. Correlation coefficients appear in ellipses and non-significant correlations are marked with an “X”.

Given these trajectories of PCA1 with age, it is not surprising that PCA1 consistently showed a positive correlation with age across populations, with the sole exception being the youngest InCHIANTI age group, consistent with the lack of large changes in PCA1 at younger ages shown above (Fig. 9). Notably, these correlations were stronger and more consistent than for the individual biomarkers, which often showed highly variable associations with age across populations (Fig. 9). The pairwise correlations between the main biomarkers determining PCA1 also varied substantially across datasets (Fig. 10). Overall, PCA1 shows stable relationships across data sets; its component markers do not.

**Fig 11:**
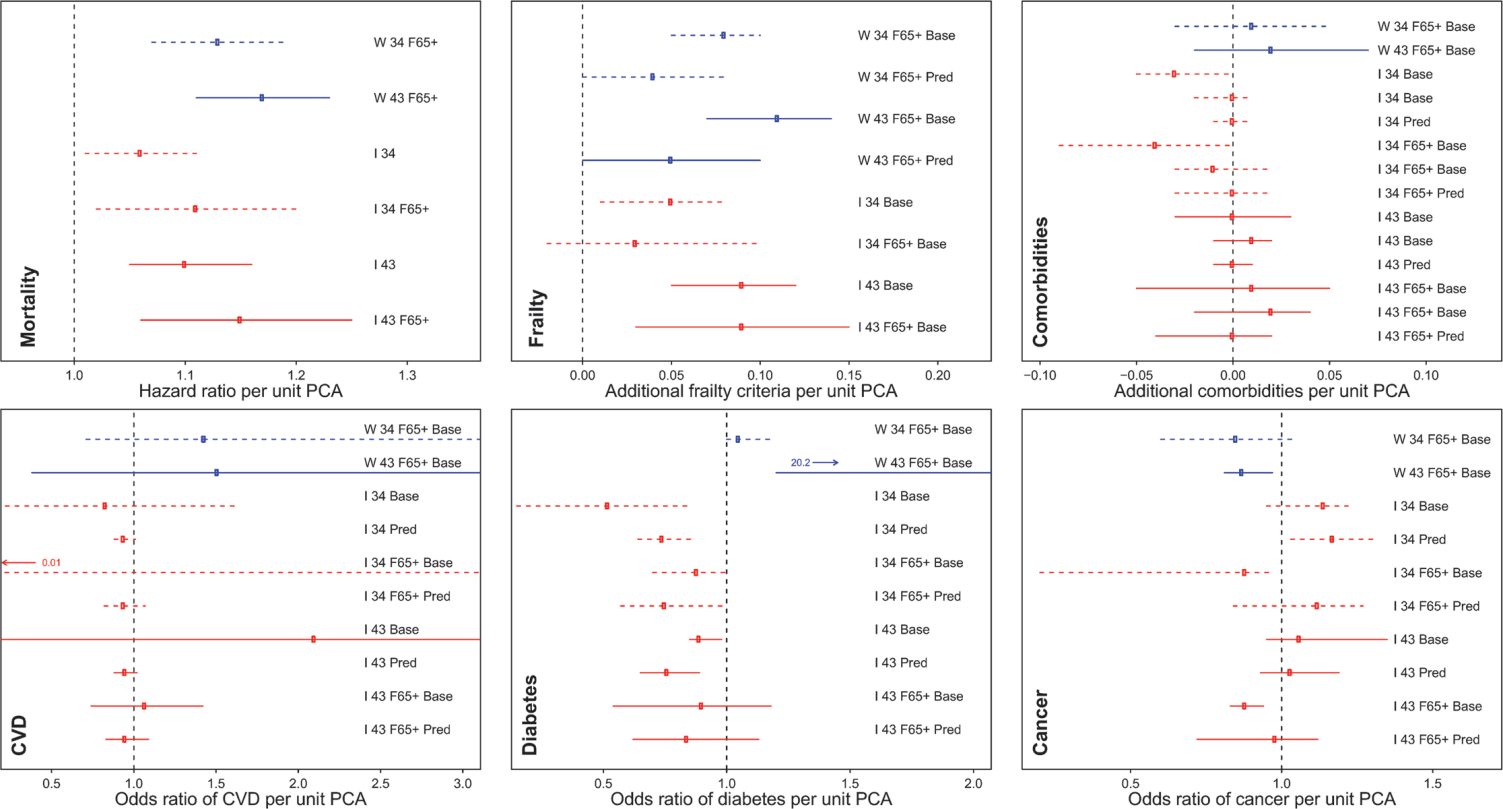
Point estimates with 95% CIs for regression models predicting health outcomes based on PCA1. Blue indicates WHAS and red indicates InCHIANTI. Solid lines are for 43-variable models and dashed lines for 34-variable models. W or I for WHAS or InCHIANTI respectively; 34 or 43 for the number of variables; F65+ if the model only included women aged 65+ (all models for WHAS); Base if the model was a cross-sectional analysis of baseline values, and Pred for models predicting a change in the parameter at the subsequent visit. Poisson regressions not shown; see S1 Results.

Additionally, we used PCA1 scores to predict subsequent health outcomes in WHAS and InCHIANTI (Fig. 11 and S1 Results; data not available for BLSA). High scores positively and strongly predicted mortality controlling for age in both datasets, with hazard ratios per unit PCA1 ranging from 1.06 to 1.18 across analyses (all *p*<0.02, Fig. 11). (Note that for all outcomes, effect sizes are per unit PCA. For odds ratios and hazard ratios, this implies exponential effects: if two individuals differ by 5 PCA units and the hazard ratio is 1.1, the individual with the higher score has 1.1^5^ = 1.6 times the risk.) Clinical frailty score (# of Fried’s frailty criteria met [24]) also showed a strong positive association with PCA1 controlling for age (Fig. 11, S1 Results), with increases of 0.05 to 0.11 frailty criteria per unit PCA1 among the 16 of 19 analyses significant at α = 0.05. Associations with both frailty and mortality remained significant with similar effect sizes even when PCA1 was generated using age-adjusted biomarkers (S1 Results). The fact that we are testing the relationship of PCA1 with six health outcomes could raise concerns that these results are due to multiple testing, particularly with the 12 additional analyses for PCAs 2 and 3. However, the clear replication of the results across datasets and analyses, with more than half of the p-values ≤0.001 (Fig. 11, S1 Results), is a clear indication of associations between PCA1 and both mortality and frailty.

In contrast, PCA1 did not consistently predict presence or incidence of cardiovascular disease, diabetes, cancer, or a comorbidity index (Fig. 11, S1 Results): although some models were significant, most were not and results went in differing directions depending on dataset, age group, and sex, such that results cannot be considered robust. In WHAS and in some cases for InCHIANTI, chronic diseases were based on self-report, introducing potential error. However, it is unlikely that this error is the full explanation for the lack of a consistent effect between PCA1 and chronic diseases. PCA2, for example, is consistently associated with diabetes (OR ≥1.35 per unit PCA2, *p*≤0.002 in all models; see S1 Results), as expected for metabolic syndrome [43]. The effect sizes for PCA1 with chronic diseases are generally small or have very wide confidence intervals, and even when results are significant they are rarely consistent with the other analyses. Accordingly, we believe that PCA1 has a complex and indirect relationship with chronic disease: these are not independent processes, but the relationships in the physiological networks are indirect enough that any detected associations depend strongly on multiple additional factors.

These results show that PCA1 is clearly associated with the aging process, increasing within individuals as they age, but also predicting mortality and frailty independently of age. The lack of clear associations with major chronic diseases also suggests that PCA1 is not a consequence of aging-related pathologies. It thus appears that PCA1 has a role in the aging process itself, though it is too early to say whether this role is merely correlational, is causal, or represents a physiological adjustment to other changes during aging. Regardless of its role, PCA1 may serve as a useful clinical biomarker of aging, providing a more stable signal than its component markers. It is easy to calculate using the reduced, 14-variable version. An Excel spreadsheet calculator and equation are provided to facilitate calculation for interested readers; see S1 Worksheet and S1 Text.

To further explore the clinical predictive value of PCA1, we compared the capacity of PCA1 to predict mortality and frailty to age alone and to age and PCA1 combined. In Cox models for mortality in WHAS, PCA1 alone had *R* ^2^ = 0.018, age alone had *R* ^2^ = 0.020, and both together had *R* ^2^ = 0.031. The hazard ratio for PCA1 with age included diminishes slightly compared to the PCA1-only model, from 1.169 to 1.134. For InCHIANTI, the same respective numbers were *R* ^2^ = 0.050, 0.098, and 0.121, with a drop in the hazard ratio from 1.33 to 1.12. For frailty in WHAS, the model form does not allow the calculation of a simple *R* ^2^, but the effect of PCA1 remains highly significant with or without control for age (*p*<0.0001), and declines from 0.146 to 0.104 frailty criteria. For frailty in InCHIANTI, the respective *R* ^2^s were 0.090, 0.185, and 0.200, and the effect size drops from 0.177 to 0.085 frailty criteria. Taken together, these data suggest that PCA1 is not particularly clinically useful in a population such as InCHIANTI that includes individuals of all ages; in this case, the age signal dominates because PCA1 varies little at younger ages. However, in a population of exclusively elderly, such as WHAS, there is less variation in age and the added value of PCA1 is substantial, both for mortality and frailty.

PCA2 strongly resembled metabolic syndrome, predicting mortality, current frailty status, CVD presence, and especially diabetes presence (S1 Results). It also predicted diabetes incidence but not CVD incidence or change in frailty status. PCA3 showed no consistent associations with health outcomes (S1 Results). Although nine of the 74 models were nominally significant at α = 0.05, this was not much higher than might be expected by chance, and the directions of the associations were inconsistent and contradictory. For example, for both cancer and diabetes in InCHIANTI, PCA3 could either be protective or a risk factor depending on which model was examined.

### Interpretation

Here, we have detected a heretofore unknown physiological axis, PCA1, which is strongly associated with multiple physiological systems, notably anemia and inflammation (positively) and calcium and albumin (negatively). We were able to detect PCA1 identically in three different cohorts, one from Italy and two from the US, as well as independently within multiple demographic sub-populations (male, female, black, white, younger, older, richer, poorer, etc.). Scores on PCA1 increase non-linearly with age (i.e., accelerate) within individuals, and they predict mortality and clinical frailty controlling for age. However, they are not consistently associated with heart disease, diabetes, cancer, or a comorbidity index, suggesting that PCA1 is directly implicated in aging, rather than indirectly through a role in chronic disease. PCA1 behaved more stably across populations than its individual component biomarkers, and was detected whether or not biomarkers were adjusted for age. PCA1 was not strongly associated with hepcidin, a hormone that regulates iron, hemoglobin, and some of the other markers associated with PCA1.

What are we to make of PCA1, and these observations about its behavior? That is, what biological phenomenon might explain our ability to detect the same multi-system axis so consistently across populations? We identified four general possibilities. First, PCA1 might represent the sum of the activities of its component markers, each of which is regulated more-or-less independently. After all, PCA1 is literally a linear combination of these markers, i.e., the sum of the levels of each of these markers multiplied by a coefficient. While this explanation is the simplest, it does not agree with many of our results. Levels of and correlations among individual biomarkers fluctuate widely across populations, as do their correlations with age. In contrast, PCA1 showed highly consistent relationships with individual biomarkers and age. In other words, PCA1 as the sum of many independent processes does not explain why the coefficients that give a weight to each process are so consistent across populations, even while the processes themselves vary.

Second, PCA1 may represent a correlation among different systems due to their correlation with some demographic or health trait, most likely age. If each of the biomarkers associated with PCA1 changed predictably with age, perhaps PCA1 was essentially a proxy for age. Again, this hypothesis was not supported by our analyses. We detected PCA1 even after adjusting the component biomarkers for age (see S1 Text for details and further validation), and PCA1 was more consistently associated with age across populations than its component markers. It is possible that PCA1 is associated with some other unmeasured demographic variable, but if this is the case the variable would have to be quite stably distributed across our three populations, and it would be hard to reconcile with PCA1 appearing more stable in its associations than its component biomarkers. We thus consider this possibility unlikely.

Third, PCA1 may represent the activity of a regulatory molecule that coordinates functioning among iron/oxygen transport, inflammation, protein transport, and calcium. It was to test this possibility that we measured the association between PCA1 and hepcidin, which was too weak to support this hypothesis. However, we cannot exclude the possibility that some unmeasured or undiscovered molecule may play a similar role, or that urinary hepcidin may not provide a good signal of functional hepcidin in the circulatory system.

The fourth possibility is that PCA1 represents a physiological regulatory mechanism that exists not as a single clear chain of regulation, but as a general property of the regulatory dynamics among a large number of molecules, a property determined by the overall structure of the regulatory relationships among the molecules. There is good theoretical reason to suspect that this is how regulatory networks should behave [7]. Structural features of networks such as redundancy and feedback loops allow the network to maintain homeostasis across a range of conditions [44], but with the result that the same physiological state can often be attained via different pathways. In turn, this means that not all possible physiological states (i.e., combinations of marker levels) are equally likely; physiology will be “channeled” to certain combinations [7]. PCA1 may represent such a channel: allowable combinations of regulatory molecules along a gradient that adjusts an important aspect of regulation. Such an interpretation is strongly in agreement with more general complex systems theory and network theory; indeed, such network structures were predicted by Kauffman over 20 years ago [7].

There are a number of reasons to believe that PCA1 does indeed represent such an emergent physiological process. Most importantly, this interpretation is consistent with all of our results. Notably (a) PCA1 is exactly replicable across populations and sub-populations; (b) PCA1 produces a more stable signal than its component markers, which vary across populations in their correlations with age and with each other; (c) PCA1 spans multiple physiological systems; and (d) PCA1 does not appear to be reducible to any single marker or variable, such as age or hepcidin or major chronic diseases, despite the large number of biomarkers measured. This interpretation is also consistent with a recent study by Matteini et al. [31], who found that heritability of aging-related PCA axes is higher than for individual variables. Lastly, our detection of metabolic syndrome as PCA2 confirms the validity of our approach in general, and suggests that metabolic syndrome may be another example of such a higher-level physiological process, if it is defined as a continuum rather than a condition that is either present or absent [28].

Whether or not PCA1 represents a higher-order physiological process, it is sufficiently stable that we can safely interpret it as a physiological phenomenon linking anemia, inflammation, and levels of albumin, calcium, ALT, and alkaline phosphatase, among others. We term this phenomenon “integrated albunemia” to reflect its multi-systemic nature, and the strong implication of measures related to anemia and albumin. Because integrated albunemia is related to inflammation it may seem similar to “Inflammaging” [4], but in InCHIANTI, where we are able to measure both [45], the correlation is quite weak (*r* = 0.17, *p*<0.0001). In fact, while inflammatory markers are consistently associated with integrated albunemia, the association is much weaker than for the anemia markers. Integrated albunemia is not obligately high when inflammation is high, and can be moderately high even when inflammation is moderately low: it is not a measure of inflammation, but rather of the confluence between anemia, albumin levels, inflammation, and several other processes. Critically, integrated albunemia is not a condition but rather a spectrum, with everyone falling somewhere along the spectrum.

### Implications

We have detected a novel physiological phenomenon, integrated albunemia, which is implicated in aging. Further study is likely to yield therapeutic and/or diagnostic advances, as well as a better understanding of aging mechanisms. The stability of the process across multiple populations suggests it is a general feature of human biology, though further study in more distinct populations would be required to confirm this.

The inability of any single marker (at least any studied) to adequately describe integrated albunemia suggests that it is a higher-order or emergent physiological process. If this is correct, there are likely to be many other higher-order physiological processes that govern important aspects of physiology, processes that we have not been looking for because they cannot be detected by isolating individual molecules or pathways. Such processes would in turn be targets of natural selection, with physiology adjusted over evolutionary time at least in part by tweaking the aspects of network structure that determine these processes [5]. Identifying gene targets acted on by selection to control key physiological processes may help elucidate how network structure determines physiological processes, and may help clarify the role of aging genes [46,47]. Detection of such system-level processes is possible using straightforward multivariate statistics such as those used here. Much of the systems biology literature has focused on mapping complex biological networks and then identifying emergent properties of these networks, or on conducting high-dimensional analyses [39,40,48,49]. Such bottom-up approaches are producing important breakthroughs, but have the disadvantage of requiring relatively exhaustive databases and knowledge of network structure. Our approach aligns more with a top-down approach [50], focused more on network function that structure. To our knowledge, this is the first study to use the correlation structure of molecules in a biological network to make functional rather than structural inferences about the network.

## Supporting Information

S1 Results Full results of 253 regression models measuring associations between PCA axes and health outcomes. The principal table is available on the “Results” tab and explanations are in the “Guide” tab. Columns A-J define the model (e.g. which PCA axis as predictor, which health outcome, type of regression model, data subsets, etc.), columns K-O give the effect size and uncertainty for the relationship between the PCA axis and the health outcome controlling for age, and columns P and Q give the sample size(s) available.
(XLSX)

S1 Text Additional methodological details and a formula related to the clinical tool. (DOCX)

S1 Worksheet A tool to calculate PCA1 for individual subjects based on existing biomarker data. This tool allows a user to input biomarker data from one or many individual patients and calculate scores on PCA1 for each patient, using the reduced set of 14 biomarkers described in the text. Additionally, explanations are included and a histogram is provided as an interpretation reference for different scores. Users should be careful to only alter text in the yellow cells; altering text in the white or green cells can cause the calculator to stop functioning. Data on all 14 biomarkers is needed to calculate a score.
(XLSX)
