# Supplementary material for: Detection of a Novel, Integrative Aging Process Suggests Complex Physiological Integration
Source: PLoS One. 2015 Mar 11;10(3):e0116489. doi: 10.1371/journal.pone.0116489 (PMC4356614; doi:10.1371/journal.pone.0116489)
Supplement: S1 Text — (DOCX) [file pone.0116489.s002.docx]

Supporting Information for

Detection of a novel, integrative aging process suggests complex physiological integration

Alan A. Cohen, Emmanuel Milot, Qing Li, Patrick Bergeron, Roxane Poirier, Francis Dusseault-Bélanger, Tamàs Fülöp, Maxime Leroux, Véronique Legault, E. Jeffrey Metter, Linda P. Fried, and Luigi Ferrucci

**Health status measures**

Health status measures were not available for BLSA. Type and availability of data varied substantially between WHAS and InCHIANTI; variables were defined as follows:

*Diabetes*

For InCHIANTI, prevalent diabetes was defined as definite (score of 1) when fasting plasma glucose level was ≥126 mg/dL, based on the American Diabetes Association 2003 criteria, or when there was use of diabetes drugs (insulin and analogues, or oral hypoglycemics). A score of 0.5 (possible diagnosis) was attributed to self-reported diabetes and treatment of diabetes through diet. Diabetes diagnosis was assumed to apply to all future visits. For WHAS, diabetes positivity was based on self-reported physician diagnosis. Thus, in InCHIANTI each individual had a score or 0, 0.5, or 1 at each time point; in WHAS, each individual had a score of 0 or 1 at baseline.

*Cancer*

For InCHIANTI, the presence of cancer was defined through self-reported malignant neoplasms (ICD-9 codes 140 through 208 inclusively, except 173). We retained only recent diagnoses, i.e. within 5 years. For follow-up visits, newly diagnosed cancers that occurred within the last three years were considered. In WHAS, the presence of cancer was defined through self-reported physician diagnosis. Thus, in InCHIANTI each individual had a score or 0 or 1 at each time point; in WHAS, each individual had a score of 0 or 1 at baseline.

*Cardiovascular disease*

For InCHIANTI, cardiovascular disease (CVD) was considered positive when at least one of the following was present: angina pectoris, myocardial infarction, congestive heart failure (CHF), stroke, peripheral arterial disease (PAD) or severe stenosis on coronary angiography. The presence of angina pectoris was defined as definite (score of 1) when there was use of organic nitrates and either one of the following: self-reported diagnosis, documentation or Rose Angina Questionnaire (ROSANG) score. If only one of medication, self-report, documentation or ROSANG score was present, a possible diagnosis was given (score of 0.5). We only retained angina pectoris diagnosed within the previous year or under current treatment. The presence of myocardial infarction was considered definite when there were signs of necrosis on electrocardiography (ECG), documented and self-reported diagnosis, aorto-coronary bypass or angioplasty. A possible score was attributed when only self-report was present. Only diagnoses within the previous year were retained. A definite score for CHF was attributed when self-report and physical exam or documentation were present or when any of those three, in addition to medication (diuretics or aldosterone antagonists and angiotensine II antagonists, angiotensin-converting-enzyme inhibitors or digitalis glycosides) or evidence in the ECG exam (presence of necrosis, atrial fibrillation in the cardiac rhythm, incomplete or complete L bundle branch blk in the intraventricular conduction, or L ventricular hypertrophy or overload), was present. A possible CHF score was given when only one of the aforementioned criteria was met. Stroke diagnosis was based on self-report, physical exam and documentation; when two or more criteria were present, a definite score was given while a possible score was attributed when only one diagnostic criterion was met. Transient ischemic attack (self-report) was considered as possible stroke. A definite score for PAD was given to ankle-brachial index (lower blood pressure of the two legs) lower than 0.9. A possible score was attributed to evidence in the physical exam, documentation or Rose PAD questionnaire. The Rose PAD questionnaire score was calculated according to responses to Rose claudication items (pain in legs while walking, standing still or sitting, pain in the calf and pain in the leg or foot at night). Diagnoses for angina pectoris, CHF, stroke, PAD and severe stenosis were adjusted across follow-up visits, as described above. In WHAS, CVD was considered positive when at least one the following was present: myocardial infarction, angina pectoris, CHF, heart disease, or stroke. All diagnoses were based on self-report. Thus, in InCHIANTI each individual had a score or 0, 0.5, or 1 at each time point; in WHAS, each individual had a score of 0 or 1 at baseline.

*Frailty*

We used Fried’s frailty criteria [[1]](#_ENREF_1) to assign a number of criteria (between 0 and 5). This measure was available at baseline in InCHIANTI and at each visit in WHAS. Fried’s frailty criteria are unintentional weight loss, fatigue, reduced grip strength, reduced physical activity, and low gait speed; fulfilling three or more of these criteria indicates clinical frailty, and fulfilling 1-2 indicates a pre-frail state. We use the number of criteria rather than frailty state in order to maximize statistical power; similar results are obtained using a dichotomous frail-non-frail outcome.

*Comorbidities*

For InCHIANTI, the number of comorbidities included definite diagnoses for the following: cancer, chronic liver disease (self-report), angina pectoris, myocardial infarction, CHF, stroke, diabetes mellitus (defined as blood glucose ≥140 mg/dL or use of diabetes drugs), chronic bronchitis or emphysema, profoundly impaired renal function (creatinine clearance assessed via Cockcroft-Gault formula ≤30 mL/min), severe stenosis, kidney failure (self-report), deep venous thrombosis (DVT) and thyroid disease. For WHAS, the number of comorbidities included any positive diagnosis for cancer, angina pectoris, myocardial infarction, CHF, stroke, diabetes mellitus, heart disease, and lung disease, all of which were based on self-reported diagnosis. Thus, for InCHIANTI each individual had a score between 0 and 13 at each time point; for WHAS, each individual had a score between 0 and 9 at baseline.

**Statistical analysis details**

*Principal components analysis (PCA)*

PCA was performed using the princomp function in R, either on the full set of 43 variables or the limited set of 34 (Table 1) using the first visit for which full data was available for any given individual. Axis loadings and variance explained were examined to assess axis interpretation and importance. It was immediately apparent that no more than the first three axes were of interest, and subsequent axes are not discussed further. Axis stability was verified using methods we have published previously [2,3]. First, analyses were stratified by non-random demographic subgroups (see “Demographic variables” above). A PCA analysis produces “loadings,” measures of how each original variable is associated with each new axis. The axes are linear combinations of the loadings; by combining the data for an individual with these loadings, one can calculate a “score” for the individual on the axis. In this way, we used the loadings from each subgroup analysis to generate new variables for the full dataset, using one randomly chosen visit per individual to avoid non-independence. For example, a woman could have a score on the axis generated using only men’s data, and vice versa. We created a correlation matrix among all the alternative versions of the axis generated using different subgroups (Fig. 6). A very strong correlation between axes calculated from independently-generated loadings indicates that the analyses in separate datasets are detecting a similar underlying process.

Second, the biological interpretation of the axes by subset was compared graphically according to the composition of their loadings, with the original variables ordered by the strength of their loadings on the full dataset and stacked in a bar graph to represent the variance of the axis they explain; subset analyses were compared in other stacked bars, retaining the original variable order from the full dataset and using randomly selected visits (Figs 1, 2, 4, 5, and 7). Homogeneity of loading order and strength across subsets would confirm that the axis identifies a similar underlying phenomenon. We also validated whether the axis structure was stable when applying PCA to age-adjusted variables (i.e., the residuals of each variable LOESS-smoothed across ages; Fig. 8).

Third, the same approaches were used across rather than within datasets [2,3]: an axis was generated from the pooled data of all three datasets, as well as separately for each dataset, and the loadings from the separate datasets were applied to the pooled dataset. For each of the first three PCA axes, a correlation matrix was generated to assess the similarity of the axes based on each of the datasets (Fig. 3). Lastly, the order of the loadings was compared across datasets, as above. After verifying the stability of the first axis, all subsequent analyses used the PCA calculated based on the full pooled dataset.

*Proper control for age*

As noted briefly elsewhere in our text, one of the most critical aspects of this analysis is full control for age. A PCA axis such as PCA1 could emerge from our data solely based on each biomarker being correlated with age, even if these variables were otherwise independent, as elegantly demonstrated by one of our reviewers. We have taken a number of steps to address this concern. Our regression models control for age using a flexible cubic basis spline (probably the best method available), and our PCA analyses were replicated on the residuals of the original variables after control for age with a flexible cubic basis spline. In the latter case, we ran extensive analyses to verify that age had been properly controlled for, including looking at the correlations of the residuals with age and examining scatterplots of age and the residuals for each bio marker.

In all cases, there was no correlation between the residual and age (|*r*|<10^-15^, *p*=1.00). Scatterplots revealed no disturbing patterns such as strange tails at the extremes, though in some cases outliers or asymmetric distributions of the variables were present. In all but two cases the spline of age explained a significant portion of the variance in the biomarker (*p*<0.0001). The exceptions were basophils (*p*=0.06) and vitamin B12 (*p*=0.88). However, in about half of cases less than 1% of biomarker variance was explained by the age spline, and in only three cases was more than 10% of the variance explained (DHEAS 11.4%, IGF-1 12.6%, and IL-6 11.2%). The mean biomarker variance explained by age was 2.6%.

While these results are relatively encouraging – there is nothing in them to explicitly suggest that we have failed to completely control for age – we cannot interpret them as proof of a sufficient control for age. Indeed, because splines are probably the best model available, there is simply no way to verify whether some small portion of the age variance remains uncontrolled.

However, we feel there are additional reasons to not be particularly concerned that PCA1 is to any large extent an artifact of uncontrolled age:

- The three biomarkers most strongly associated with age (IGF-1, DHEAS, and IL-6) are relatively weakly associated with PCA1, ranking 12^th^, 15^th^, and 17^th^ among the 43 biomarkers. If PCA1 were largely a proxy for age, all three should be within the top 10.
- If PCA1 were an artifact of age, most of its signal should disappear after controlling it (i.e., the full post-PCA axis) for age. This was clearly not the case in our original analyses.
- Controlling PCA1 for age with a linear or a spline model produced similar results, as indicated in our original manuscript. If the results were much better with a spline model, we would have more cause to worry that even a spline model is not sufficient; the fact that there is little improvement from linear to spline suggests that age is not hard to control.
- Age does not explain a large portion of the variance in the biomarkers (~2.6%, compared to about 10% for PCA1), but many of the biomarkers are relatively strongly correlated with each other. It is thus highly unlikely that the first axis of a PCA would be due to age.
- PCA is a linear combination of variables. Because the splines are both (a) non-linear and (b) different for each of the 43 biomarkers, even imperfect control for age with splines would be likely to weaken the overall signal for age within the 43 markers, unless the non-linearity in age signals AND the error in spline estimation are highly similar from one biomarker to another. That is, an error in spline control for age might mean that biomarker 1 has slightly underestimated levels at ages 66-68 and slightly overestimated levels at 72-74; biomarker 2 would be unlikely to have the same errors, but might have overestimated levels at ages 55-63 and underestimated levels at 83-84. The chance that such errors would together result in a coherent age signal across 43 biomarkers is minimal.

*Prediction of health status*

For the 43-variable analysis in InCHIANTI, some of the biomarkers (DHEAS, IGF-1, estradiol, TSH, folate, and vitamin B12) were only available at baseline, but outcome measures were available longitudinally. We wished to verify the 34-variable results with the 43-variable dataset because the inflammatory markers (IL-6 and CRP, only available in the 43-variable set) loaded well on PCA1. To achieve this, we imputed these missing variables as subsequent time points based on age, age^2^, sex, and age-sex interaction. While the behavior of imputed variables has not been properly validated in this context, all the imputed variables loaded only weakly on PCA1 and thus should have had minimal influence on the results. Indeed, the analyses with imputed variables were highly consistent with other analyses (Supporting Results).

**A simplified version of PCA1 for clinical use**

-0.46*(Hemoglobin-13.49)/1.4 + -0.41*(Hematocrit-40.35)/3.89 + -0.31*(Albumin-23.47)/26.43 + -0.30*(RBC-4.44)/0.467 + -0.30*(Iron-85.42)/29.14 + -0.29*(Albumin:Globulin-1.434)/0.247 + 0.26*(log(RDW)-2.62)/0.086 + -0.21*(MCH-30.46)/2.06 + -0.21*(MCHC-33.43)/1.165 + -0.19*(log(ALT)-2.95)/0.518 + 0.18*(log(CRP)-0.932)/1.088 + -0.15*(Calcium-9.39)/0.456 + 0.11*(Platelets-236.5)/68.13 + 0.077*(log(Alk. Phosph.)-4.59)/0.495

This formula is based on the 14 variables the most strongly associated with PCA1, chosen also so as to best represent the different physiological systems implicated and to be cheap and easy to measure in clinic. Each variable is log-transformed (if necessary), then its mean is subtracted and it is divided by its standard deviation. This ensures that all variables are normally distributed and on the same scale, with mean=0 and standard deviation=1. Then each transformed variable is multiplied by the loading generated from the PCA analysis, and the sum is the value of PCA1. We provide an Excel spreadsheet calculator to facilitate calculation by clinicians or others, including a histogram of values observed in our data set to help interpret the meaning of a given value of PCA1 (Supporting Worksheet).

**Supporting References**

1. Fried LP, Tangen CM, Walston J, Newman AB, Hirsch C, et al. (2001) Frailty in Older Adults: Evidence for a Phenotype Journal of gerontology Series A, Biological sciences and medical sciences 56: M146-M157.

2. Cohen AA, Dhingra N, Jotkar R, Rodriguez P, Sharma V, et al. (2010) The Summary Index of Malaria Surveillance (SIMS): a stable index of malaria within India. Population Health Metrics 8: 1.

3. Dusseault-Bélanger F, Cohen AA, Hivert M-F, Courteau J, Vanasse A (2013) Validating metabolic syndrome through principal component analysis in a medically diverse, realistic cohort Metabolic Syndrome and Related Disorders 11: 21-28.
